# Recent Development of Self-Powered Tactile Sensors Based on Ionic Hydrogels

**DOI:** 10.3390/gels9030257

**Published:** 2023-03-22

**Authors:** Zhen Zhao, Yong-Peng Hu, Kai-Yang Liu, Wei Yu, Guo-Xian Li, Chui-Zhou Meng, Shi-Jie Guo

**Affiliations:** 1 State Key Laboratory for Reliability and Intelligence of Electrical Equipment, School of Mechanical Engineering, Hebei University of Technology, Tianjin 300401, Chinayw16@hebut.edu.cn (W.Y.); guoshijie@hebut.edu.cn (S.-J.G.); 2 Hebei Key Laboratory of Smart Sensing and Human-Robot Interaction, School of Mechanical Engineering, Hebei University of Technology, Tianjin 300401, China

**Keywords:** ionic hydrogels, self-powered sensors, piezoelectric, tactile sensors

## Abstract

Hydrogels are three-dimensional polymer networks with excellent flexibility. In recent years, ionic hydrogels have attracted extensive attention in the development of tactile sensors owing to their unique properties, such as ionic conductivity and mechanical properties. These features enable ionic hydrogel-based tactile sensors with exceptional performance in detecting human body movement and identifying external stimuli. Currently, there is a pressing demand for the development of self-powered tactile sensors that integrate ionic conductors and portable power sources into a single device for practical applications. In this paper, we introduce the basic properties of ionic hydrogels and highlight their application in self-powered sensors working in triboelectric, piezoionic, ionic diode, battery, and thermoelectric modes. We also summarize the current difficulty and prospect the future development of ionic hydrogel self-powered sensors.

## 1. Introduction

A flexible tactile sensor is a device that can bend and deform along with the surface it is applied to, enabling it to detect pressure, touch, and other physical interactions in a more natural and intuitive way. In recent years, the scientific community has shown a keen interest in flexible tactile sensors due to their potential in the advancement of emerging human interactive technologies, including but not limited to artificial skin-like sensors, wearable or implantable devices, and user-interactive ones [1]. Hydrogels are one of the most suitable materials for constructing flexible tactile sensors due to their unique properties. Hydrogels are polymeric materials with a unique three-dimensional network structure that allows them to absorb and retain large amounts of water, resulting in distinct physical and chemical properties [2]. By incorporating conducting components into hydrogel matrices, conductive hydrogels can be constructed. Electronic conductive hydrogels have been prepared by embedding different electronic conductive materials (such as conductive polymers [3], metal nanoparticles/nanowires [4], liquid metals [5], MXenes [6,7], and carbon-based materials [8]) into the hydrogel matrix. Despite their potential advantages, inorganic materials often exhibit distinct chemical and mechanical properties compared to biological tissues. This can pose significant challenges when developing wearable devices based on inorganic materials, such as poor contact between the device and skin and unreliable signal acquisition. On the other hand, hydrogels have been endowed with ionic conductivity due to the polymer network and water molecules in them. The hydrogel’s water molecules may preserve the same chemical and physical properties as liquid water because the mesh size of the polymer network exceeds the size of a water molecule by a significant margin, measuring approximately 10 nm [9]. This opens up the possibility of creating high-quality ionic conductive hydrogel materials, in which conductivity is provided by the movement of free ions acquired from polyelectrolytes, electrolytes, and even ionic liquids dissolved in water [10]. Due to their ionic conductivity, flexibility, and tissue similarity, ionic hydrogels show greater potential as materials for wearable devices compared to electronic conductor-based ones, which are susceptible to the deterioration of conductive networks [11].

**Figure 1:**
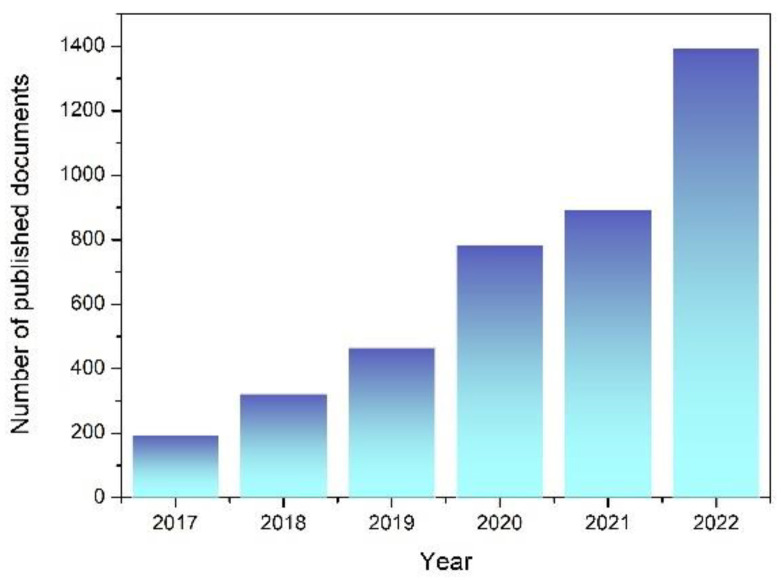
Number of published papers on the https://app.dimensions.ai/ database with the search of “hydrogel for self-powered tactile sensor” (the data were accessed on 1 February 2023).

**Figure 2:**
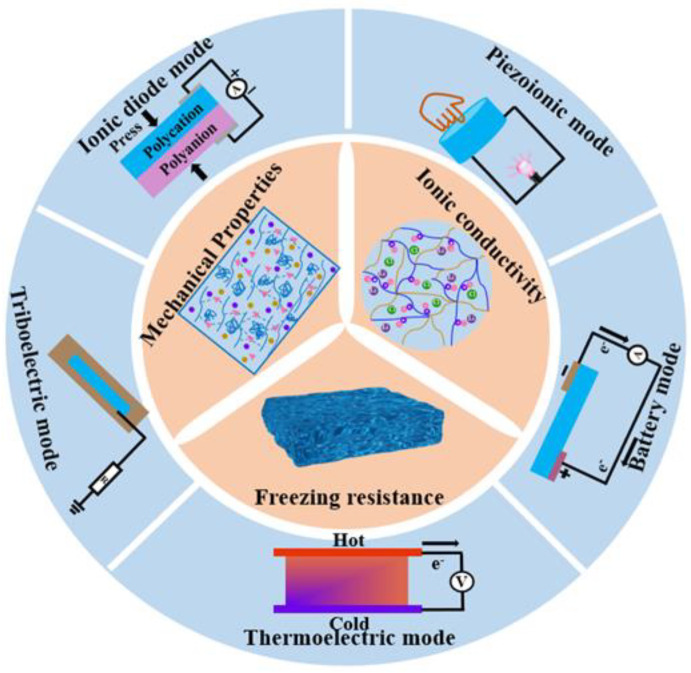
Schematic of the unique properties and modes of the ionic hydrogel self-powered tactile sensors.

Ionic hydrogels of various kinds have recently been created for tactile sensors, which generally depend on two different types of traditional sensing mechanisms. One type of flexible mechanical sensor uses resistive- or capacitive-based sensing principles to translate changes in particular electrical characteristics of resistors and capacitors into the variation of external mechanical stimuli [12,13,14,15]. For example, we have reported the design and manufacture of a flexible pressure sensor with a porous ionic hydrogel. The sensor developed by using double-layer capacitors and a micro-structured electrolyte has stable performance and can be used for finger pressing, carotid pulse measurement, finger bending, and other applications. Yang et al. developed a wearable resistivity-type sensor using a chitosan-poly (hydroxyethyl acrylamide) double-network hydrogel that is resilient, antifatigue, and freezing-tolerant [16]. This kind of flexible tactile sensor is suitable for identifying stationary or slowly varying mechanical stimuli. However, the power requirements of these sensors necessitate the use of an additional power source, which inevitably increases the volume of the sensor and compromises its portability. For practical applications, it is necessary to develop self-powered tactile sensors by integrating ionic conductors and a portable power source into a single sensor. Therefore, considerable effort has been focused on creating integrated devices that are self-powered [17]. The development of self-powered tactile sensors based on hydrogel materials demonstrated a dynamic growth in the research arena. Figure 1 illustrates how the development grows rapidly each year. The power source issue can be alleviated by integrated-circuit devices that combine hydrogel-based sensors with micro capacitors or batteries, but the energy density of these micro power sources is limited. Regular external power supply charges are still required [18]. Moreover, these devices are less practical and fail to provide the convenience that people expect from wearable technology due to the limited flexibility of the power sources. The other type of sensors, based on piezoionic, triboelectric, ionic diode, thermoelectricity, or battery mode sensing principles, have extremely low power consumptions and may independently provide electric signal outputs. Figure 2 shows the required properties and operating modes of ionic hydrogels for sensors.

Recent advances in flexible tactile sensors have been reviewed in terms of materials, devices, and applications. For example, Li et al. reviewed the developments of conductive hydrogels for flexible strain sensors [10]. Scaffaro et al. [19] provided the most recent achievements in high-performance ionic tactile sensors applied to intelligent systems. Zhao et al. [20] summarized post-treatment techniques and the development of appropriate electrical and mechanically durable interfaces of hydrogel sensors. Ying et al. [11] reviewed cutting-edge skin-like hydrogel technologies for wearable electronics, soft robotics, and energy harvesting. Liu et al. [21] provide an overview of the latest developments in wearable sensors based on transparent and stretchable hydrogels, and they focus on their materials, designs, and various applications. Rahmani et al. provide a comprehensive review of hydrogel-based wearable strain sensors, with a perspective on their unique features, strain-sensing capabilities, and potential applications [22]. Alternatively, some reviews explore the usage of innovative conductive materials [23,24]. However, the development of self-powered tactile sensors using ionic hydrogels has rarely been systematically reviewed. In this review, we focus on the basic properties of ionic hydrogels for self-powered sensors. On this basis, we present a summary of ionic hydrogels developed for self-powered sensors based on piezoelectric, triboelectric, thermoelectric, and battery mode principles. We highlight their significant benefits and promising application prospects in the field of flexible tactile sensors. In addition, we discuss the potential challenges and future prospects of self-powered sensors based on ionic hydrogels. This review exclusively focuses on ionic hydrogels, without considering other ionic gels. However, based on the same principle, ionic gels can also be utilized for self-powered tactile sensing. Ultimately, the aim is to shed light on the remarkable benefits and promising application possibilities of ionic hydrogel-based self-powered sensors.

## 2. Properties of Ionic Hydrogels

A flexible tactile sensor should be highly conductive, have strong mechanical characteristics, and be long-lasting and resistant to freezing. In order to design flexible electronic devices, it is crucial to design ionic hydrogels with high conductivity, superior mechanical characteristics, and freezing resistance (Figure 2). In recent years, scientists have made significant progress toward endowing hydrogels with a wide range of adaptable and distinctive properties needed for flexible touch sensors, including ionic conductivity, mechanical qualities, and freezing resistance.

### 2.1. Ionic Conductivity 

Due to their 3D framework structure and continuous water phase, hydrogels offer a large number of pathways for ion movement. This opens up the possibility of synthesizing good ionic conductive hydrogel materials. Ionic hydrogels have so far been created for flexible electronic devices using a variety of ionic conductive materials, such as electrolytes [25], polyelectrolytes that constitute networks of hydrogels [26], and ionic liquids [27,28] that dissolve in the solvent phase. They increase the hydrogels’ resistance to freezing in addition to providing conductivity. In the meantime, ions typically improve the mechanical characteristics of hydrogels by interacting with polymer chains through processes such as the salting-out effect or coordination [29].

One easy and quick method to make an ionic conductive hydrogel is by directly doping the hydrogel with soluble inorganic salts such as LiCl [30] and NaCl [31]. Because hydrogels have a high water content and a microporous structure, resolvable salts can disperse easily, allowing the hydrogels to conduct ions. Polyelectrolytes are polymers containing ionic moieties in their repeating units [32], and they can be crosslinked chemically or physically to produce polyelectrolyte hydrogels. Polysaccharide-based polymers (alginate [33], chitosan [34], carrageenan [35]), polypeptide/protein-based polymers (gelatin [36], collagen [37]), and synthetic polymers (polyacrylic acid [38], poly(sulfobetaine) [39], carboxymethyl cellulose [40]) can be used to create polyelectrolyte hydrogels. Ionic liquids (ILs) are molten salts with melting temperatures under 100 °C that are made up of organic cations and organic or inorganic anions. In terms of conductivity and stability, ILs are a promising class of small-molecule liquids for providing free ions. By adjusting the loading amount and creating an appropriate network structure for hydrogels, ionic conductive materials can impart proper conductivity to hydrogels.

Guo et al. [41] obtained poly(amidoxime)/polyethyleneimine (PAO/PEI) hydrogel through hydrogen bond interactions. PAO with amidoxime groups has outstanding ionic adsorption properties, which results in an ultrahigh ionic conductivity of 19.1 S m^−1^ in 6 M LiCl for the PAO/PEI hydrogels. This hydrogel with high ionic conductivity is expected to have a broad application prospect in wearable sensing devices.

Yang et al. [42] synthesized a zwitterionic hydrogel through the random copolymerization of a zwitterionic monomer (SBMA) and 2-hydroxyethyl acrylate (HEA) with the addition of LiCl salt. The anionic and cationic counterions present on the zwitterionic chains promote the dissociation of LiCl, leading to a high room-temperature ionic conductivity of 14.6 S m^−1^.

Yao et al. [43] developed an ionic conductive hydrogel platform through a simple one-step method by incorporating cellulose nanofibrils (CNFs) into a crosslinked network of phenylboronic acid-ionic liquid (PBA-IL) and acrylamide. By increasing the PBA-IL content, the ionic conductivity of the hydrogels was enhanced from 0.92 ± 0.10 to 6.94 ± 0.21 mS cm^−1^, rendering them attractive soft sensing materials.

Ionic hydrogels generally exhibit relatively low conductivity due to the delicate balance between ion concentration and water content in the hydrogels. To fulfill the need for improved performance in sensing devices, continuous attempts have been made to produce hydrogels with enhanced ionic conductivity. The distinct attributes of the ionic hydrogels will serve as a crucial reference guide for the development of the next generation of wearable electronics and electronic skin.

It should be emphasized that ionic hydrogels conduct electrical charges through the migration of mobile ions and the “ion–electron” conversion on the electrode surfaces. As a result, AC mode is typically used to test the electrical resistance or impedance of ionic conductors. When an ionic hydrogel is in contact with a metal surface, mobile ions and electrons come together to create an electric double layer (EDL) at the interface. The EDL performs the role of a capacitor by coupling the electronic current in the metal and the ionic current in the hydrogel [44].

### 2.2. Mechanical Properties

Excellent mechanical strength of soft conductive materials is essential for wearable tactile sensors in order to maintain excellent structural integrity and reliable signal monitoring capability under complex loads and repeated large deformations. Since ionic hydrogels often have weak but adaptable mechanical characteristics and excellent stretchability, they have shown to be an attractive alternative for the creation of flexible tactile sensors. As a result, significant efforts have been devoted to improving the mechanical properties of these hydrogels, especially for applications such as ionotronic skin (i-skin).

The most commonly employed method for producing tough and highly conductive hydrogels involves creating a double-network (DN) structure that utilizes reversible crosslinking [45]. A DN is made up of two networks: a second network that is soft and ductile and a first network that is rigid and brittle [46]. Sun et al. [47] developed a DN ionic conductive hydrogel that possesses impressive mechanical properties and conductivity. The hydrogel was created by immersing a virgin gellan gum/gelatin composite hydrogel in a mixed solution of Na_2_SO_4_ and (NH_4_)_2_SO_4_. This hydrogel exhibits adjustable Young’s modulus (ranging from 0.08 to 42.6 MPa), fracture stress (0.05 to 7.5 MPa), fracture stretch (1.4 to 7.1), high fracture toughness (up to 27.7 kJ m^−2^), and superior ionic conductivity (up to 11.4 S m^−1^ at f = 1 kHz). The enhancement in mechanical properties of the DN gel is attributed to the introduction of chain-entanglement crosslinking points by ${SO}_{4}^{2-}$ in the gelatin network and electrostatic interaction crosslinking points by Na^+^ in the gellan gum network. Meanwhile, soaking in salt solutions is an important way to strengthen DN conductive hydrogels.

Polyols were employed as effective physical crosslinking agents to enhance the strength and toughness of ionic hydrogels. Peng et al. [48] described the fabrication of a PVA-NaCl-glycerol hydrogel through the incorporation of glycerol and NaCl into an aqueous solution of PVA, followed by storing at room temperature for 2.0 h. Benefiting from the hydrogen bonding and salting-out effect, the PVA-NaCl-glycerol hydrogels demonstrated exceptional toughness, exhibiting a tensile strength of 0.57 (±0.02) MPa and an elongation at break of 575 (±29)%. Moreover, these hydrogels exhibited remarkable conductivity, reaching as high as 92.5 (±0.18) mS/cm.

Ionic hydrogels show more suitable mechanical adaptability to epidermal or muscle tissues than conventional polymeric materials [49]. Hence, there is a need to dedicate further efforts towards advancing the development of ionic hydrogels in order to enhance the performance of tactile sensors.

### 2.3. Freezing Resistance

Generally, a traditional hydrogel composed of a pure hydrophilic system inevitably freezes at temperatures below zero; the ion transmission is blocked, and the hydrogel is hardened and brittle, which seriously limits its application in the low temperature range [50,51]. Therefore, ionic hydrogel-based flexible tactile sensors with reliable antifreezing performance have received a lot of attention. In order to solve these problems, the current antifreezing strategy is to incorporate different antifreezing additives, such as ionic compounds, natural biopolymers, and organic/aqueous solvents into the polymer network, which help to change the water–ice phase equilibrium at different stages of ice nucleation and growth [52,53,54]. 

Inorganic salts such as CaCl_2_, ZnCl_2_, and LiCl and ionic liquids or poly(ionic liquids) are widely used as water freezing inhibitors to be introduced into hydrogels to increase their low-temperature resistance. For example, Wang et al. [55] synthesized a zwitterionic composite hydrogel with antifreezing and water retention properties, which are attributed to the incorporation of LiCl as an electrolyte and antifreeze agent. Electrostatic dissociation of LiCl with the zwitterions contributed to the high conductivity of the composite hydrogel (7.95 S m^−1^) and excellent antifreeze performance, reaching as low as −45.3 °C. At the same time, due to the presence of salt ions, the composite hydrogel was observed to retain 97% of its initial water content after exposure to air (25 °C, 55% RH) for one week. 

Another approach is to introduce organic solutions such as ethylene glycol (EG), sorbitol, DMSO, and glycerol into the hydrogel. For example, Yu et al. [51] prepared high-ionic-conductivity hydrogels with excellent antifreeze and dehydration resistance by immersing cellulose nanofibril (CNF)-reinforced hydrogels in CaCl_2_/sorbitol solution for solvent replacement. The synergistic effect of sorbitol and CaCl_2_ makes the hydrogels exhibit excellent freezing resistance, dehydration resistance, and ionic conductivity. Strong hydrogen bonds between water and sorbitol molecules prevent the formation of ice crystals and the evaporation of water, giving CS-NC hydrogels very-low-temperature resistance to −50 °C and excellent dehydration resistance, with weight retention in excess of 90%. Wang et al. [52] introduced a water-retaining antifreeze ionic conductive hydrogel composed of silk fibroin, ionic liquid, water, and inorganic salts. Silk fibroin (SF)/1-ethyl-3-methylimidazolium acetate (EMImAc)/H_2_O/KCl-based hydrogel electrolytes work well at temperatures as low as −50 °C and after prolonged storage outdoors. 

The inorganic salts used to create the antifreezing hydrogels have good ionic conductivity, but when added in large quantities, the inorganic salts can compromise the hydrogels’ mechanical strength. On the other hand, in the presence of organic solvents, the ionic conductivity of the hydrogel will be greatly reduced due to the reduced ion dissociation in organic solvents and limited ion mobility with enhanced crosslinking density. In addition, organic solvents are known to be environmentally hazardous and raise health and safety issues [53]. Recently, Zhang et al. [54] introduced and demonstrated a comprehensive crosslinking approach for 4,9-dioxo-5,8-dioxa-3,10-diazadodecane-1,12-diyl diacrylate (EGINA) crosslinked double-network hydrogels that exhibit inherent antifreezing properties. The antifreezing mechanism is solely derived from the formation of tightly bound water with networks via hydrogen bonds and the confinement of the water in the tightly crosslinked DN structure. In summary, antifreezing hydrogels are capable of retaining their ionic conductivity and mechanical properties even at low temperatures, making them ideal for use in extreme environments. With the continuous development of novel materials and advanced crosslinking strategies, antifreezing hydrogels are expected to achieve even higher performance and expand their range of applications.

## 3. Different Modes of Ionic Hydrogel Self-Powered Sensors

Due to their distinctive benefits, including great stretchability, designable conductivity, and superior compliance, ionic hydrogels have been extensively explored and employed in the field of tactile sensors. The majority of wearable flexible tactile sensing systems require bulky wiring and an external power source, which restricts the development of smart electronic devices. While integrated-circuit devices that incorporate small capacitors/batteries can address this issue, they are less convenient and often struggle to meet the comfort needs of users when it comes to wearable technology. Therefore, the development of self-powered tactile sensors would be highly advantageous for creating portable and comfortable electronic devices.

In this section, we review recent developments in self-powered tactile sensors based on ionic hydrogels operating under different sensing modes, including piezoionic, triboelectric, thermoelectric, and battery modes.

### 3.1. Triboelectric Mode

**Figure 3:**
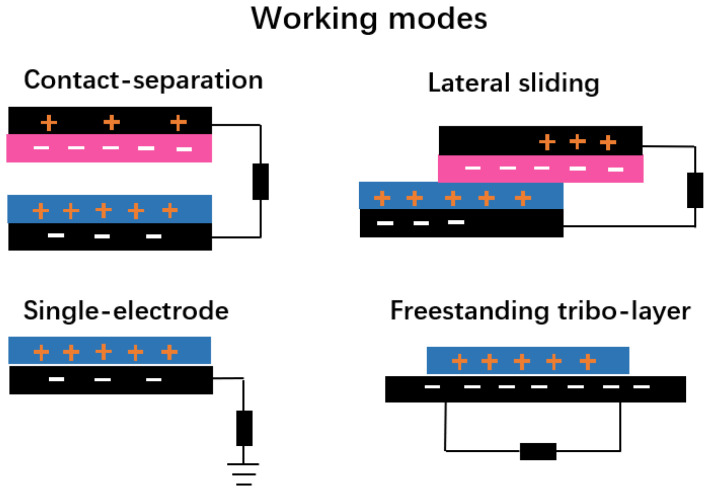
Working modes of TENGs.

Triboelectric nanogenerators (TENGs) can convert ambient mechanical energy into valuable electricity based on the mechanisms of contact electrification and electrostatic induction [56]. Four working modes are commonly used in TENGs, including vertical contact-separation mode, lateral sliding, freestanding tribo-layer mode, and single-electrode mode (Figure 3). Due to the differing electron attraction properties of two materials, when they come into contact, electrons can move from one to the other. An alternating current is created when two materials continuously come into contact or separate as a result of external stimulation. TENGs have shown tremendous advantages in self-powered sensors [57,58]. Besides triboelectric materials, electrodes also play a significant role in the operation of TENGs. Among the diverse electrodes of TENGs, a hydrogel with high transparency, excellent mechanical properties, tunable ionic conductivity, biocompatibility, and environmental friendliness might be a good candidate for the application of a TENG for flexible and wearable tactile sensor applications [59,60].

**Figure 4:**
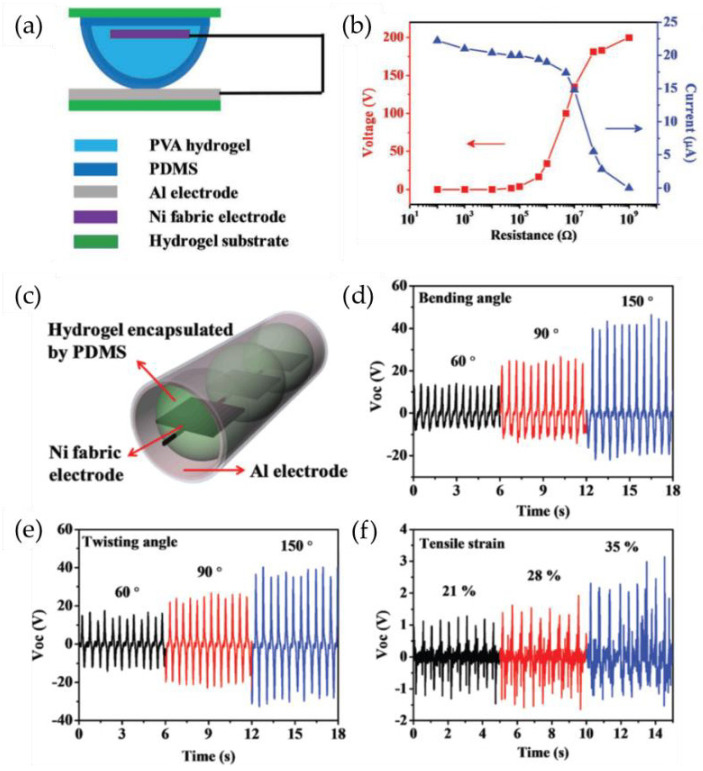
(**a**) Schematic of the hydrogel-based triboelectric generator. (**b**) Output voltage and current versus the resistance of the external loads. (**c**) Schematic diagram of the Hydrogel-TENG in a tube shape, along with the open-circuit voltage generated by (**d**) bending, (**e**) twisting, and (**f**) tensile strains. (Reprinted with permission from [61].)

Xu et al. [61] developed a PVA hydrogel-based triboelectric nanogenerator (Hydrogel-TENG) system for the first time; this system can serve as a self-powered sensor to detect human motions. The PVA hydrogel was fabricated using the freeze–thaw method. The Hydrogel-TENG structure comprises two components: a top contact layer with a hemispherical shape and a bottom contact layer with a flat structure. The top contact layer incorporates a nickel fabric electrode and a hemisphere-shaped PVA hydrogel, which is encapsulated by a PDMS film and serves as the triboelectric layer. The bottom layer consists of an aluminum foil that is affixed to the surface of the hydrogel substrate (Figure 4a). The PVA hydrogel was used as an elastic structural material due to its high elasticity. By leveraging the hemispherical design morphology, the Hydrogel-TENG is able to alter the contact area between the PDMS and aluminum foil in response to external forces. This leads to triboelectrification between the foil and the nickel fabric within the hydrogel. The resulting device is capable of producing an open-circuit voltage of 200 V and a short-circuit current of 22.5 μA. At a load resistance of 10 MΩ, the peak power output of the device is approximately 2 mW (Figure 4b). Then the hemisphere-shaped top contact layers were connected in series and wrapped with a bottom contact layer to form a tube-shaped Hydrogel-TENG (Figure 4c). The PVA-based Hydrogel-TENG is capable of acting as a self-powered human motion sensor by capturing biomechanical energy from stretching, bending, and twisting (Figure 4e,f).

**Figure 5:**
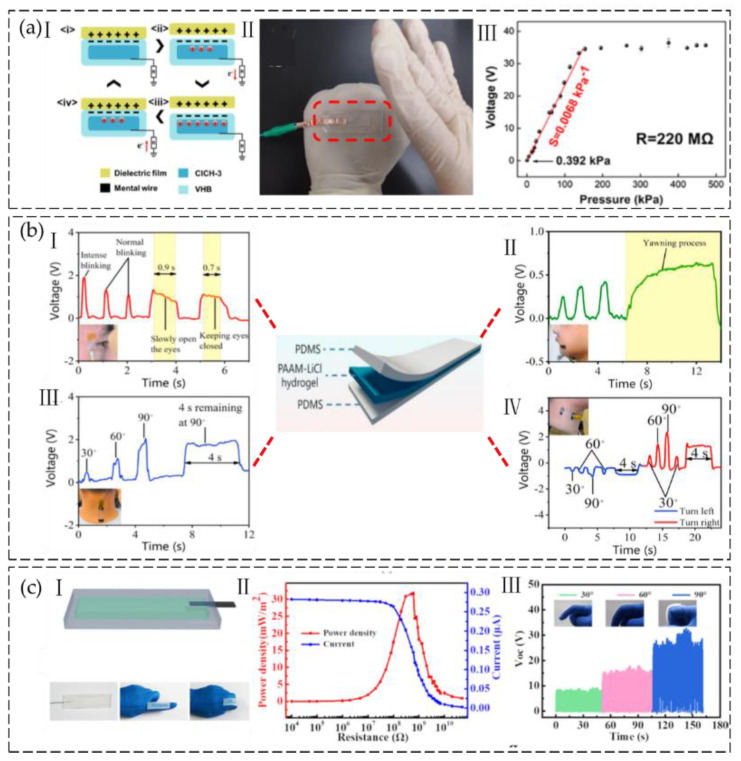
(**a**) Cellulose hydrogel for flexible sensor. I. Schematic working mechanism of CNH TENG. II. A transparent hydrogel TENG tapped by fingers. III. Various peak amplitudes of voltage across the resistor (220 MΩ) with different pressures applied. (Reprinted with permission from [63].) (**b**) Use of a hydrogel-based TENG for driving fatigue monitoring. I. Eye-closure movement detection. II. Yawning detection. III. Head-turning movement detection. IV. Vertical bending angle detection. (Reprinted with permission from [65]). (**c**) Self-Powered Smart Arm Training Band Sensor. I. Schematic diagram of the SA−Zn hydrogel TENG and the photographs of the SH-TENG in its original and foldable state. II. Output Performance of the SH-TENG. III. The voltage outputs of the SH-TENG under bending of the finger. (Reprinted with permission from [66].)

The most typical design for a hydrogel-based TENG consists of two layers of elastomers surrounding a conductive hydrogel connected to the ground, adopting the single-electrode triboelectric mode. For the TENG, these elastomer layers serve as dielectric layers [62]. Electrons move from the ground to the electrode as contact-separation cycles proceed between the elastomer and another external dielectric substance. In a study by Hu et al. [63], cellulose hydrogels that are flexible, transparent, and conductive were produced by regenerating chemically cross-linked cellulose in an aqueous solution of NaCl. NaCl in the hydrogel matrix played a dominant role in modulating the properties of the cellulose hydrogel, resulting in excellent mechanical properties (tensile strength and elongation at break of 5.2 MPa and 235 %), conductivity (4.03 S/m), transparency over 94% at 550 nm, and low-temperature tolerance down to −33.5 °C. A piece of cellulose/NaCl hydrogel (CNH) sample was sandwiched by two layers of VHB. Latex was used as an external triboelectric layer (Figure 5(aI)). The cellulose hydrogel-based triboelectric nanogenerator was connected with an external load of 220 MΩ to construct a self-powered tactile/pressure sensor with a sensitivity of 0.0068 kPa^−1^ and a pressure detection limitation of about 0.392 kPa (Figure 5(aII,III)). In addition, glycerol was incorporated into the cellulose hydrogel to enhance its antidrying characteristics and increase its tolerance to low temperatures, as low as −63.7 °C. The cellulose hydrogel-based TENG sensor exhibited high optical transmittance at 550 nm, with a value of 97%, as well as excellent tensile strength of 6.8 MPa, elongation at break of 220%, and conductivity of 0.72 S m^−1^. Further analysis revealed that the sensor’s sensitivity to pressure was calculated to be 0.0103 kPa^−1^, with a pressure detection limitation of approximately 16.8 Pa [64].

Lu et al. [65] developed a hydrogel-based TENG for monitoring driving fatigue. This innovative system can detect exhaustion and distraction by generating distinct electrical signals in response to abnormal movements made by the driver. The TENG device comprises polydimethylsiloxane (PDMS) and ionic hydrogel layers serving as friction layers and electrodes, respectively. Ionic hydrogels were made of polyacrylamide (PAAM) and LiCl. A PAAM/LiCl ionic hydrogel was sandwiched between dielectric layers of PDMS. Briefly, uncured PDMS was poured into an acrylic mold. After it was cured, an Al tape was attached to PDMS as a wire, and then hydrogel pre-solution was dropped onto PDMS and semi-cured. Subsequently, a pre-fabricated layer of PDMS film was placed over the hydrogel layer, and the entire mold was then cured in an ultraviolet chamber to solidify the hydrogel. The hydrogel-based TENG operated in a single-electrode mode, and the resultant device was affixed to the driver’s skin, which served as the TENG’s second dielectric material. Detection of driver fatigue was based on an assessment of three parameters, namely the duration of blink intervals, the percentage of eyelid closure over time, and the frequency of yawning, against predetermined thresholds. The duration of blinks was also utilized to provide the driver with a timely reminder (Figure 5b).

Sheng et al. [66] constructed a TENG using a double-network polymer ionic conductor sodium alginate/zinc sulfate/poly acrylic-acrylamide (SA−Zn) hydrogel, which exhibited outstanding stretchability (>10,000%), high transparency (>95%), and good conductivity (0.34 S·m^−1^). The SA−Zn hydrogel TENG (SH-TENG) was obtained by encapsulating the SA−Zn hydrogel with ecoflex films (Figure 5(cI)). The power density of the SH-TENG can reach a maximum of 32 mW·m^−2^ at an external resistance of 0.6 GΩ (Figure 5(cII)). The self-powered sensor was assembled by encapsulating the SA-Zn hydrogel with silicone rubber to form a sealed sandwich structure. The output voltage of the SA-Zn TENG showed a direct linear correlation with the applied force, indicating its potential as a smart arm training band sensor for real-time monitoring (Figure 5(cIII)).

**Figure 6:**
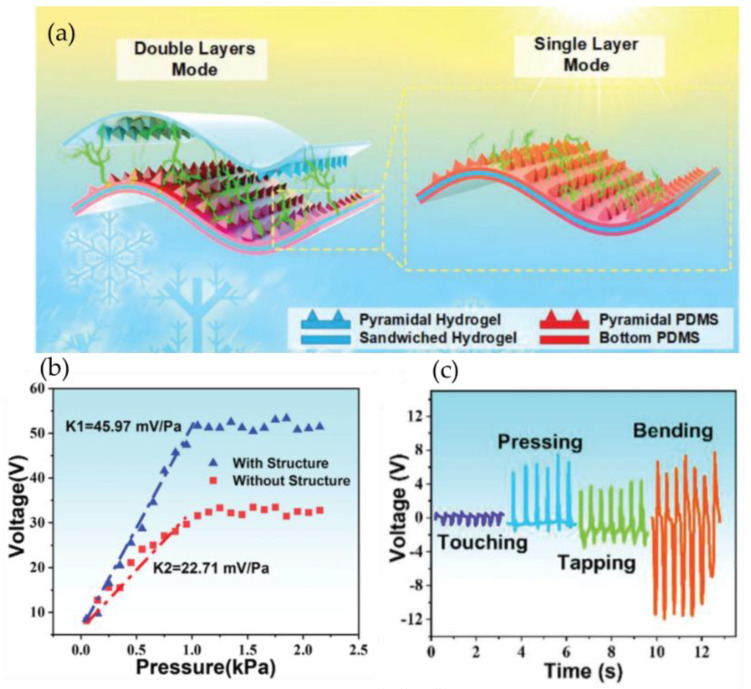
(**a**) Schematic diagram of the DE-THS and SE-THS. (**b**) Open-circuit voltage of both the plain SE-THS and the micro-pyramid-patterned SE-THS varied with different pressure. (**c**) Output signals of DE-THS under touching, pressing, tapping, and bending (reprinted with permission from [68]).

The hydrogel TENGs discussed above worked in the single-electrode mode using ionic hydrogel encapsulated with triboelectric material as one electrode. Ionic hydrogel-based TENGs working in the contact-separation working mode with two electrodes have been described [67]. Ionic hydrogels can also be used as triboelectric layers. Tao et al. [68] fabricated an ionic hydrogel by immersion treatment of micro-pyramid-patterned polyacrylamide (PAAm)/carrageenan DN hydrogel with LiBr solution. The ionic DN hydrogel was employed as both the triboelectric layer and flexible electrode. The hydrogels can be utilized in various working principles, including the double-electrode triboelectric hydrogel sensor (DE-THS) and the single-electrode triboelectric hydrogel sensor (SE-THS) (Figure 6a). Because of the effectively enlarged contact areas and higher triboelectric effect, the presence of micro-pyramid-patterned PDMS and hydrogel greatly increases the sensitivity of the sensors. In the case of the SE-THS sensor, a chemigum layer was employed as the lower charge affinity material to contact the PDMS layer. The researchers observed that the output voltage of the SE-THS was considerably higher than that of the DE-THS due to the significant electron affinity differential between the chemigum layer and PDMS. This can be attributed to the chemigum losing electrons more rapidly than the hydrogel during the triboelectric process. The SE-THS exhibits an outstanding peak power density of 20 μWcm^−2^ and can achieve a high sensitivity of up to 45.97 mV Pa^−1^. Moreover, the flexible micro-pyramid-patterned hydrogel sensors have been successfully integrated into joints and masks to detect human physiological motions, thereby offering efficient and convenient means for healthcare monitoring (Figure 6b,c). 

Recently, there has been significant progress in the development of hydrogels in TENGs, particularly in the area of self-powered tactile sensor applications [69,70,71,72,73,74]. Similar to other types of TENGs, hydrogel-based TENGs also face many problems and hurdles. Hydrogels have a lower conductivity than typical flexible metal electrodes. The methods used to provide hydrogels with ionic conductivity, such as immersing them in salt solutions or creating stable charge channels, still require further improvement. Currently, the output of hydrogel-based TENGs is less than that of other flexible TENGs, limiting their self-power efficiency. As a result, additional research is necessary to advance these methods towards commercial viability.

### 3.2. Piezoionic Mode

**Figure 7:**
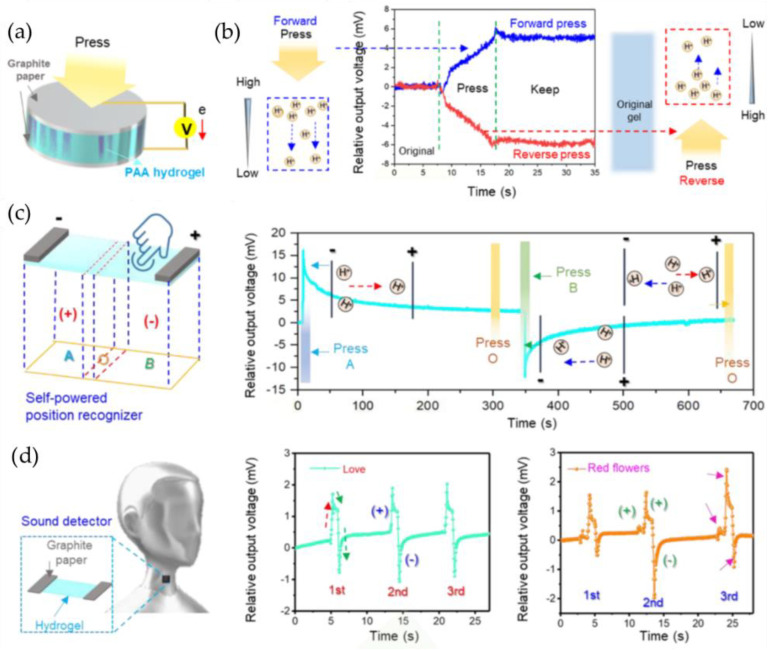
(**a**) Schematic diagram of PAA hydrogel-based sensor. (**b**) The working principle of PAA hydrogel converting mechanical energy into electrical energy. (**c**) A schematic diagram of the self-powered position recognizer and the corresponding variations in the output voltage of the detector, in response to pressing at different locations on the sensor. (**d**) Application of the PAA hydrogel-based sensor as a sound detector. (Reprinted with permission from [78].)

It is a well-established fact that the transportation of a single ion, be it a cation or an anion, generates electrical power. When subjected to pressure gradients, ionic hydrogels can produce ionic currents directly, thus offering a piezoionic method of sensing [75]. Piezoionic sensors work by generating an ion concentration gradient under pressure (Figure 7a). The deformation of the hydrogel causes an increase in the local ion concentration, and the directional diffusion of ions occurs due to the difference in ion concentration [76]. This happens when a single polarity of ions is streamed through the polymer matrix or when one polarity of ions is preferentially carried over the counterions, resulting in a net charge imbalance [77]. 

In polyelectrolyte hydrogels, the network structure of hydrogel blocks the free movement of charged chains in the framework. The great difference between free-moving ions and fixed charged chains brings the possibility of conversion between mechanical energy and electrical energy of compressed hydrogels. A self-powered sensing platform that performs electromechanical conversion was created by Pan and his colleagues [78], using a polyelectrolyte hydrogel. They employed a polyacrylic acid (PAA) hydrogel as a model system that can ionize H+ in water due to its -COOH group. They found that the H^+^ concentration gradually declines from the upper to lower surfaces of the hydrogel due to the compression behavior of the PAA hydrogel, which causes a gradual decrease in compression degree (relative displacement) from top to bottom. Since there is a gradient in the concentration of H^+^, H^+^ in the hydrogel will diffuse downward and provide an output voltage (Figure 7b). The fundamental principle behind power generation is the direct diffusion of H+. PAA hydrogels are capable of converting static or low-frequency (~0.05Hz) pressure (0.05-5N) into DC voltage/current. At a pressure of 5 N, the voltage output of the hydrogel can reach 6 mV and the current can reach 2 μA. Direction-dependent self-powered sensing is possible with polyelectrolyte hydrogels. A sensor for self-powered orientation (position) detection was created based on this characteristic (Figure 7c). The self-powered PAA hydrogel sensor that has been obtained can also serve as a voice recognizer (Figure 7d). The concept of mechanical energy–electrical energy conversion can also be applied to other electrolyte hydrogels. 

**Figure 8:**
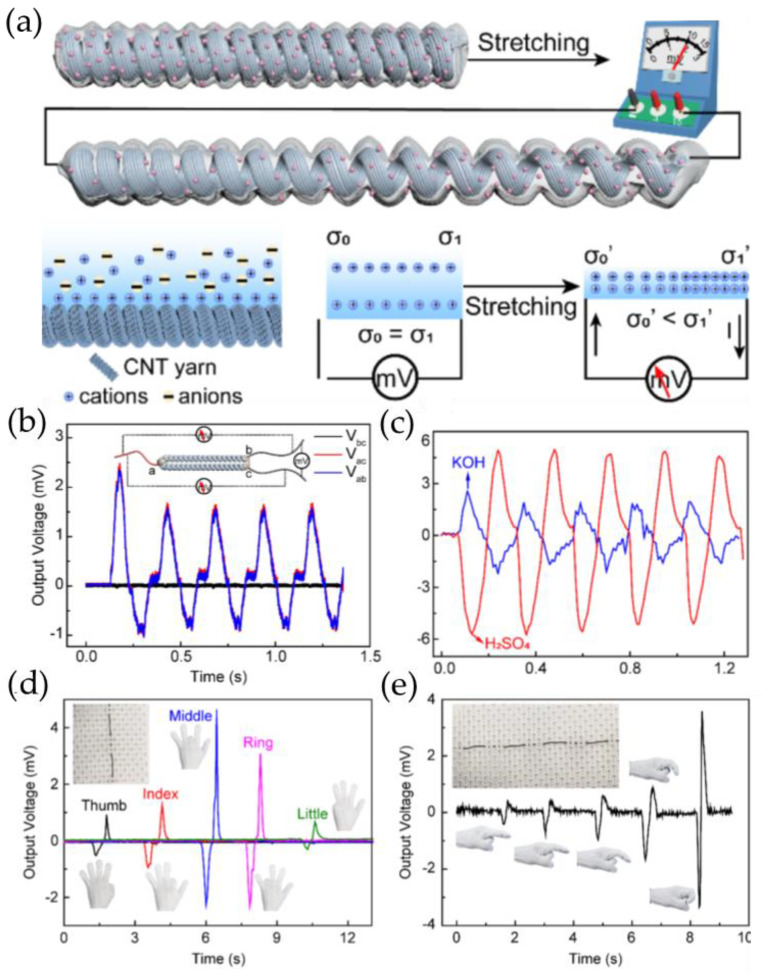
(**a**) Schematic illustration of the mechanism of the voltage generated by dynamic structure-nonuniform-induced ion squeezing. (**b**) Output voltage resulting from the two-end-symmetry-stretch tests conducted on the coiled CNT@PVA/H_2_SO_4_ yarn, with the middle portion secured while both ends are simultaneously stretched. (**c**) The output voltage signal of CNT@PVA/H_2_SO_4_ and CNT@PVA/KOH yarns with reversed phase. (**d**) The output voltage signal of the sensors attached on each finger of a hand with different finger motions. (**e**) Signal generated by the bending of an index finger at varying degrees. (Reprinted with permission from [79].)

Li and his colleagues [79] developed stretchable piezoionic yarns, which are made up of coiled CNT yarns that are wrapped in hydrogel electrolytes. (PVA/H_2_SO_4_/PVA/KOH/PAAm/NaCl). The sensor has the ability to produce a voltage gradient throughout its length, without requiring any additional counter electrodes. When subjected to asymmetric stretching, the yarn can generate sensitive and recognizable voltage signals (ranging from 4 to 15 mV) with minimal noise (0.024 mV) between its two ends. The generation of electricity along the conductive piezoionic yarn is explained by a mechanism of dynamic structure-nonuniform-induced ion squeezing. When in contact with ionic hydrogels, the surface of the coiled MWCNT yarn develops an EDL. When the coiled yarn is at rest, the EDL remains stable and uniformly charged. However, upon stretching, the structural changes in the yarn are unable to keep pace with the transmission of force, owing to structural hysteresis which results in an asymmetric change in surface area throughout the coil length, generating a potential difference along the yarn (Figure 8a). The potential gradient produced by the asymmetric stretch is responsible for producing the stretch voltage. These authors found that the polarization of the generated voltage is related to the type of absorbed electrolytes (Figure 8c). The amplitude of the voltage is highly responsive to tensile stretches over a broad spectrum of frequencies (0.1−10 Hz) and strains (1−80%). The yarn can be used to distinguish gestures and other human joint motions (Figure 8d,e).

**Figure 9:**
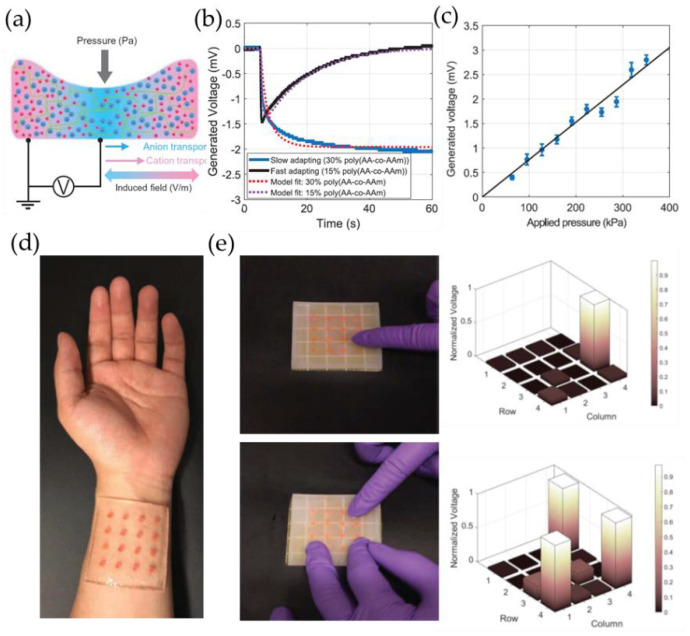
(**a**) The schematic illustrates a hydrogel undergoing indentation, with differential ionic displacement and field observed. The smaller red cations are transported through the green polymer chain network more rapidly than the blue anions, creating a charge imbalance and generating an electric field. (**b**) Voltage response of PAAm hydrogel upon step compression at 20 kPa. (**c**) Peak voltage produced in relation to the pressure being applied. (**d**) A wrist-mounted 16-element piezoionic mechanoreceptor array. (**e**) Photograph and related normalized voltage bar plot of the piezoionic mechanoreceptor array that detects single and multiple touches. (Reprinted with permission from [75].)

Dobashi and colleagues [75] conducted an indentation experiment to investigate the molecular underpinnings of the piezoionic effect and to explore its potential applications in sensing (Figure 9a). When pressure is applied to the hydrogel, the pressure will disperse the ions in the liquid at different speeds, thus generating electrical signals. Smaller positive ions move faster than larger negative ions. This will lead to uneven distribution of ions, resulting in an electric field. The transient response time depends on the polymer content. Polyacrylamide hydrogels containing sodium chloride were fabricated at various polymer contents and film thicknesses to demonstrate varying transient responses. Hydrogels that have a lower polymer content exhibit a faster response, which can be attributed to the increased permeability of the hydrogel matrix, which increases the flow in the pores carrying protons, thus resulting in a faster rise (Figure 9b). An artificial mechanical sensor consists of a poly(AA-*co*-AAm) hemisphere with a diameter of 2 mm and a height of 2 mm. The hemisphere is surrounded by a PAAm plane swelled with 0.1 M KCl. A single mechanoreceptor unit has a pressure sensitivity of 8 μV/kPa (Figure 9d). When tested under single-touch and multitouch conditions, a 4 cm by 4 cm sensor array was able to detect a voltage change of approximately −10 mV in response to a finger press of around 100-gram force. It is worth noting that this voltage change is superimposed on the Donnan potential of −50 mV (Figure 9e).

Piezoionics provides soft, self-powered, and biocompatible sensor technologies. The force–voltage coupling effect makes ionic hydrogels promising candidates for sensor application. However, the slow transfer rate of ions and the low rate of recovery of polymers in ionic hydrogels frequently result in a prolonged response time (>1 s) before reaching a steady state [79]. For this technology to be effectively utilized as a self-powered pressure sensor in electronic skin, there needs to be a thorough investigation of the electromechanical coupling mechanism in the future. 

### 3.3. Ionic Diode Mode

Ionic diodes made of a pair of polycation and polyanion ionic conductors have recently gained attention as potential components for self-powered ionic skins [80,81]. In the interface of the two ionic hydrogel layers, a depletion layer (or ionic double layers) will be formed, and the self-induced potential of ionic diodes is stabilized. The bilayer’s deformation (pressure, compressive strain, and stretching strain), as well as the rediffusion of mobile ions caused by the deformation, can change the potential. Self-powered sensors made of ionic diodes can effectively generate electricity from low-frequency mechanical inputs such as human motion.

**Figure 10:**
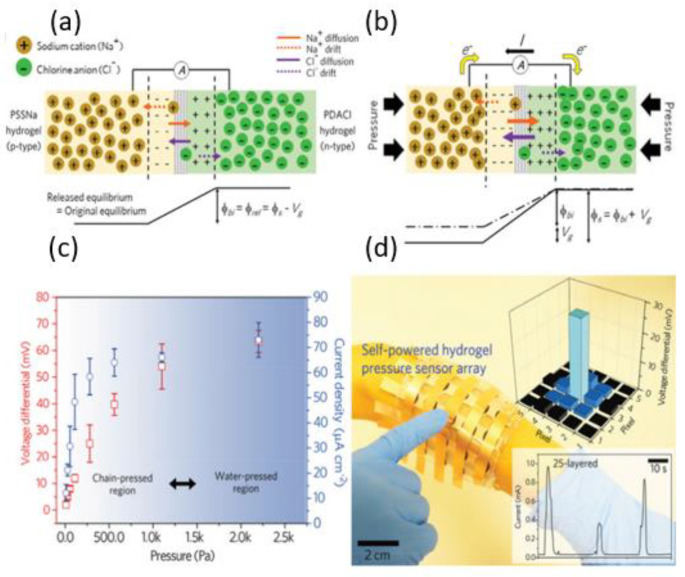
(**a**) The electrical behavior and response of the ionic diode including potential diagram. (**b**) The electrical behavior and response of the ionic diode under external mechanical stress. (**c**) Voltage and current output generated at various external pressures. (**d**) Picture of an arm-wrapped self-powered hydrogel tactile sensor array. Insets: (top panel) voltage signal mapping obtained by pushing the center pixel. (Reprinted with permission from [82].)

Zhang et al. [82] developed a hydrogel ionic diode where the two hydrogel layers were formed by blending agarose with ionomer sodium polystyrene sulfonate (PSSNa) and polydiallyldimethylammonium chloride (PDACl), respectively. When PSSNa is dissolved in the hydrogel, it exists as an anionic polystyrene sulfonate backbone (PSS−) and mobile Na+ cation, while PDACl dissociates into a cationic poly(diallyldimethylammonium) (PDA+) backbone and a mobile Cl− anion within the hydrogel. Mobile cations and anions create a depletion region, and the fundamental mechanism underlying the hydrogel ionic diode involves an increase in the built-in potential across the depletion region in response to mechanical pressure (Figure 10a,b). Within the range of 0.01–2.2 kPa, the output voltage of the self-powered sensor increased from 2 mV to 60 mV as the pressure increased (Figure 10c). A 5 × 5-pixel ionic array was used as a prototype self-powered sensor array showing excellent promise of the ionic diode for tactile sensing (Figure 10d).

**Figure 11:**
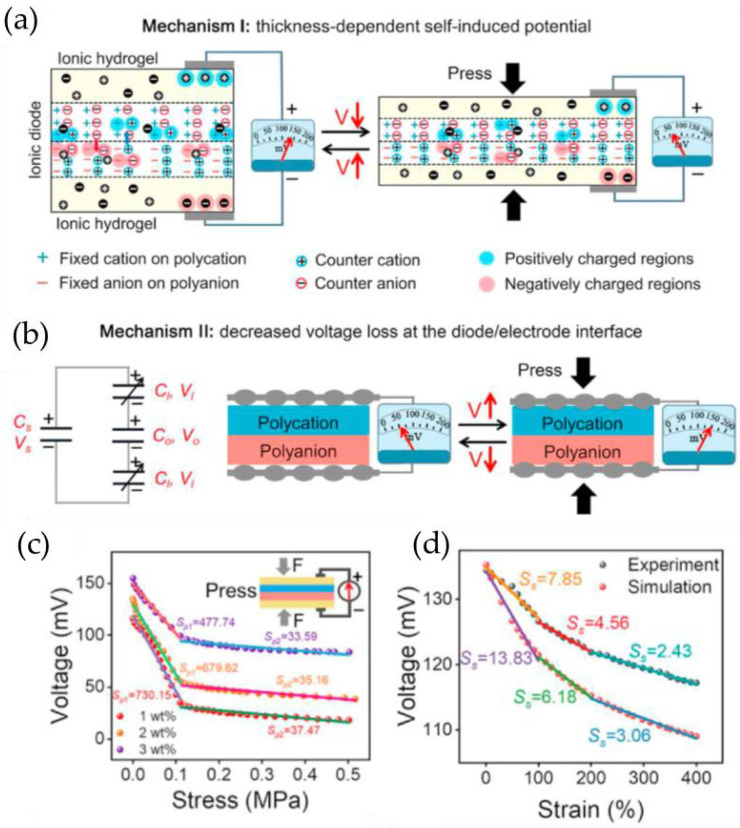
(**a**) Thickness-dependent self-induced potential of ionic diode. (**b**) Decreased voltage loss at the diode/electrode interfaces in response to pressure. (**c**) Output voltage and sensitivity of self-powered pressure sensors as a function of pressure. (**d**) Output voltage and sensitivity of self-powered sensors as a function of strain. (Reprinted with permission from [83].)

Du et al. [83] developed a flexible approach to build self-powered sensors using stretchable ionic diodes. These sensors consist of two ionic hydrogel electrodes with a hydrogel-based ionic diode in between. Polyacrylamide (PAM)/methacrylated sodium alginate (MSA), PAM/methacrylated chitosan (MCS), and PAM/NaCl hydrogel sheets were used as polyanion hydrogel, polycation hydrogel, and hydrogel electrodes of the ionic diode, respectively. The authors noted that the devices based on ionic diodes contain two mechanisms for converting mechanical energy into electrical energy: the thickness-dependent self-induced potentials, and the variable potential losses at the interfaces between the diode and the electrodes (Figure 11a,b). By using ionic hydrogel electrodes, they produced ionic diode-based self-powered sensors based on the thickness-dependent built-in potentials of ionic diodes. The sensor is capable of detecting pressures as low as 50 Pa, with a pressure sensitivity of 679.62 mV/MPa for pressures under 0.1 MPa and 35.16 mV/MPa for pressures over 0.1 MPa. It can generate consistent waveforms in response to pressures ranging from 50 Pa to 5000 Pa (Figure 11c,d).

**Figure 12:**
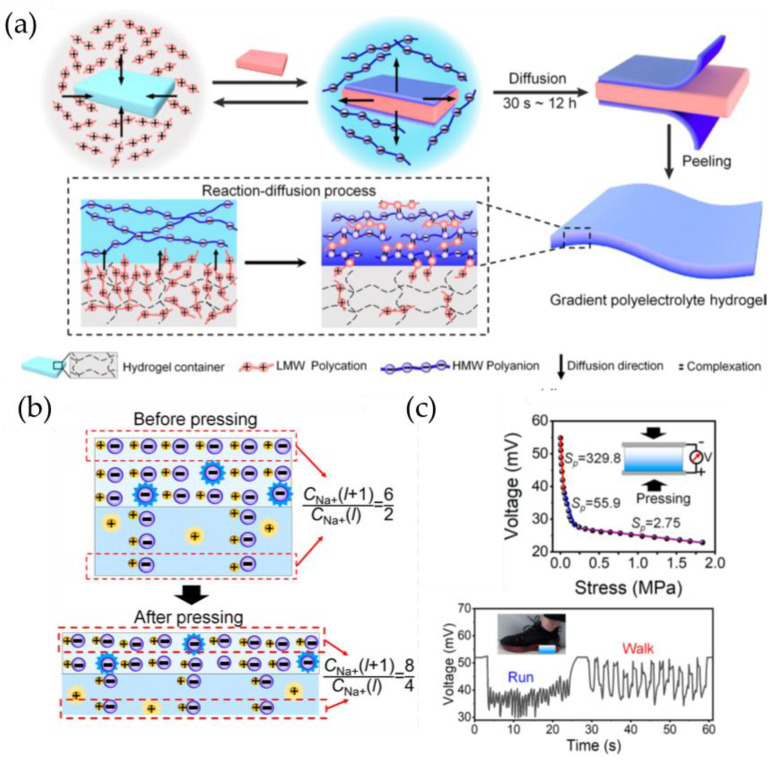
(**a**) Synthesis mechanism of hydrogel-based GPMs. (**b**) Schematic illustration of pressure-sensing mechanism. (**c**) The voltage produced by self-powered i-skins while experiencing gradually increasing pressure, as well as in response to walking and running motions. (Reprinted with permission from [84].)

Xia et al. [84] created self-powered, human-like ionic skins (i-skins) based on membranes made of gradient polyelectrolytes (GPMs). The GPMs were constructed using a hydrogel-assisted reaction–diffusion technique and had charged groups that were distributed gradientially across the membranes. By attaching the upper and lower sides of the hydrogel-based GPMs to two Pt electrodes, the self-powered ionic skins were obtained. The mechanoelectric conversion mechanism of the sensor is based on the thickness-dependent self-induced potential (Figure 12a). The pressure sensitivity is ∼329.8 mV/MPa at a pressure lower than 50 kPa and decreases to 2.75 mV/MPa over 200 kPa (Figure 12b). The sensors can be used to monitor both low-frequency walking motions (∼0.35 Hz) and high-frequency running motions (∼1.4 Hz) (Figure 12c). The self-powered sensors can also detect minute vibrations, such as the periodic pulse and vocal cord vibration.

The ionic diodes can effectively generate electricity from low-frequency mechanical stimuli and self-power the sensor. Nonetheless, devices based on ionic diodes have some limitations, including unfavorable delamination between diode components and a complex preparation process for gradient ionic hydrogels. Additionally, there is still ongoing debate regarding the mechanoelectric conversion mechanism used by self-powered sensors based on ionic diodes.

### 3.4. Battery Mode

Mostly, ionic hydrogels work under non-Faradaic processes, with no matter or charge crossing the interface between a hydrogel and a metal, where mobile ions and electrons form an electric double layer (EDL) [9]. On the other hand, hydrogels have been widely used as electrolytes for solid-state batteries, in which the insertion and extraction of ions on the two electrodes can maintain a consistent ionic concentration in the hydrogel electrolyte. It is expected that using ionic hydrogel sensors directly as the electrolytes of batteries will provide benefits such as maintaining a stable chemical composition within the sensors during current transport, as ions are inserted and extracted on two electrodes [85]. When an external strain or pressure is applied, the resistance of the hydrogel electrolyte will change due to deformation. This will alter the electronic or current signal, which can then be converted into a voltage signal by connecting it to a resistor.

**Figure 13:**
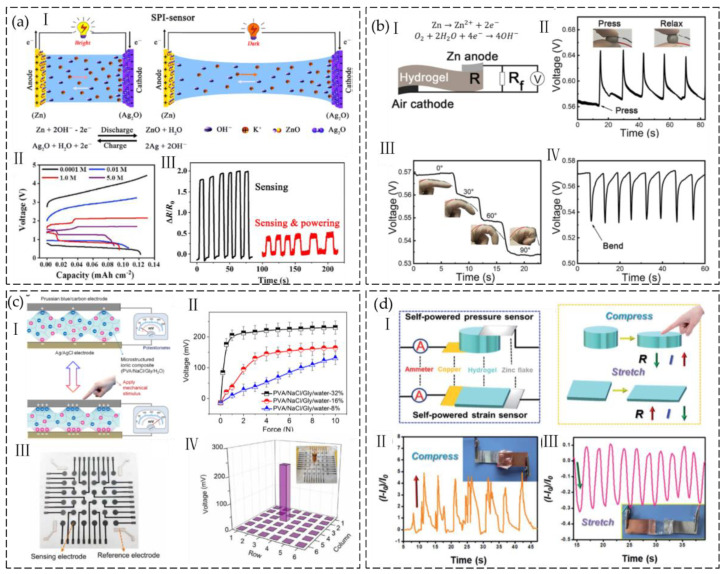
(**a**) I. Self-powered strain-sensing mechanism of the self-powered ionic hydrogel-based sensor (SPI). II. Charge–discharge curves of SPI sensors. III. Relative resistance changes of SPI sensor in response to elbow-joint motions in no-load state and in the state of powering a watch. (Reprinted with permission from [85].) (**b**) I. Schematic illustration of pressure-sensing mechanism. II. The voltage changes when pressure is applied and released. III. Voltage variation of the sensor in response to finger bending. IV. Cyclic voltage variations with finger bending angles ranging from 0° to 90°. (Reprinted with permission from [86].) (**c**) I. Mechanism of the potentiometric mechanotransduction. II. Responsive characteristics of mechanotransducers with varied Gly content. III. Photograph of the single-electrode-mode e-skin with 6 × 6 sensing pixels. IV. Response behaviors of the e-skin. (Reprinted with permission from [88].) (**d**) I. Schematic illustration of the self-powered pressure and strain sensor. II. Relative current variations of the sensor in response to compression. III. Relative current variations of the sensor in response to strain. (Reprinted with permission from [89].)

Zhang et al. [85] utilized the Zn-Ag battery system to provide sensors with self-powering capabilities. The ionic hydrogels were prepared by immersing PAM hydrogels directly into KOH solutions. To assemble the sensor, the two sides of the ionic hydrogel were attached to a Zn electrode and an Ag electrode. After the sensors were charged, an Ag_2_O layer was produced on the Ag electrode. The spontaneous oxidation of Zn and reduction of Ag_2_O can power the sensor when the circuit from two electrodes is complete (Figure 13(aI)). The charge/discharge studies indicate that the sensors can provide stable output voltage (Figure 13(aII)). The sensitivity of the sensors was calculated to be 0.95 at a small strain of 50% and increased to 8.9 as the strain was increased to 2000%. It was discovered that the sensors need a rather low ion concentration to function with enough sensitivity. The sensor can work steadily for more than 3 hours after charging for around 7 minutes. Furthermore, the dual functions of sensing and self-powering enable the sensors to power a watch while simultaneously detecting the bending motions of the elbow joint (Figure 13(aIII)).

Wang et al. [86] developed a self-powered strain sensor by connecting a hydrogel battery to a fixed resistor. The hydrogel battery was formed by sandwiching a TA crosslinked gelatin hydrogel doped with Ag nanowires (Ag NWs) between a zinc electrode and an air electrode (Figure 13(bI)). The constructed hydrogel battery was able to convert the chemical energy of the Zn-air galvanic cell into electrical energy, resulting in an open circuit voltage of 1.15 V (Figure 13(bII)). The voltage output of the fixed resistor terminal responds to pressure and strain signals owing to the dependence of hydrogel’s resistance on the elongation (Figure 13(bIII,IV)). The strain sensor is flexible, self-healing, and self-powered, thanks to the hydrogel’s high stretchability (≈1600%), rapid self-healing ability (within 0.65 s), and high self-healing efficiency (95%). The sensor was capable of continuously and reliably detecting finger bending degrees, allowing for the monitoring of joint motions.

By exploiting the common metallic corrosion phenomena, Liang et al. [87] present electrochemical and self-powered pressure sensors. Two electrodes are used to create potential differences using the inherent variations in the corrosion activities of various metals (zinc, aluminum, copper, etc.). Ionic hydrogel electrolytes of PVA/NaCl/Gly with microstructures prepared by a mesh-molding method are then used to encode external mechanical stimulations into the potential difference variations. The microstructure hydrogel was designed to increase the electrode/electrolyte interfacial contact area variation when pressure was applied to the device. The resulting pressure sensors with C/Zn electrode combination have extremely low power consumption, high sensitivity (182.3 mV/N or 3.64 mV/kPa), and quick response and recovery times (57.1 ms and 70.0 ms, respectively). They also have good reproducibility (2000 cycles), high performance tunability, and—most significantly—a unique ability to monitor static or slowly varying mechanical stimulations in a self-powered manner. When combined with a machine learning algorithm, the proposed electrochemical pressure sensors showed tremendous promise for high-accuracy (99.05%) recognition of speech. a variety of human physiological processes, and body motions.

Wu et al. [88] select two active electrode materials (i.e., PB/carbon and Ag/AgCl) that can generate a potential difference when brought into contact with a PVA/NaCl/Gly/water ionic hydrogel electrolyte. Efficient and successful regulation of the voltage output by externally applied force is dependent on the high-impedance ionic composite and the microstructure (Figure 13(cI)). The mechanotransducers exhibit a sensitivity of 205.5 mV/N in the force range of 0 to 1 N (the contact area of the applied force is ≈16 mm^2^). Subsequently, there is a decrease in signal variation (2.3 mV/N) within the force range of 1 to 10 N (Figure 13(cII)). The response and recovery times of the mechanotransducers are measured to be 71 and 106 ms, respectively. The potentiometric mechanotransducers can detect both static and low-frequency dynamic mechanical stimuli within the range of 0.5 to 2 Hz with self-generated voltage output and exhibit extremely low power consumption and highly tunable sensing behavior. The authors demonstrated a flexible e-skin with 6 × 6 sensing pixels based on hydrogel sensors in a single-electrode mode, demonstrating its ability to differentiate the magnitude of the applied force (Figure 13(cIII,IV)).

Wang et al. [89] designed and fabricated a self-powered hydrogel-based sensor inspired by a fruit battery. It is well known that when metal electrodes are put into fresh fruit, electricity can be produced. This is ascribed to a redox reaction that occurs between the acidic liquid inside the fruit and the electrode. To replicate fruit’s capacity for self-power, they combined biocompatible malic acid (MA) and Ca^2+^ in a PVA hydrogel. The hydrogel exhibits several desirable properties, including high stretchability (approximately 830%), a Young’s modulus similar to that of skin (~30 kPa), good electrical conductivity (~5.3 S m^−1^), and resistance to drying and freezing (~47.92 °C). The researchers designed self-powered pressure and strain sensors using a PVA-based hydrogel as the electrolyte and thin copper and zinc foils as the electrodes. The sensors can produce voltage outputs of approximately 0.55 V and current outputs through redox reactions (Figure 13(dI)). Once pressured or stretched, the resistance of the hydrogel will vary accordingly; thus, the sensor responds to pressure and strain signals through output current changes (Figure 13(dII,III)). 

Li et al. [90] developed an electrochemical-driving self-powered strain/pressure sensor utilizing a hydrogel composed of polyacrylamide/carboxymethyl chitosan/LiCl(PAAM/CMC/LiCl). The hydrogel featured great stretchability (a significant strain of 640% with a tensile strength of 67 kPa), antidrying and antifreezing capabilities (−48.27 °C), self-healing capacity, and good electrical conductivity (0.56 S/m). A flexible zinc-ion battery is constructed by directly assembling a PAAM/CMC/LiCl hydrogel with Zn and MnO_2_ electrodes (ZIB). A fixed resistor was connected in series with the flexible battery to serve as the signal output terminal, allowing for the detection of observable sensing signals. The resistance change of the PAAM/CMC/LiCl hydrogel was then converted into a voltage change across the fixed resistor. These sensors can detect a variety of human activities, including joint movements, subtle swallowing, and low-magnitude sound waves.

The self-powered sensors assembled by using ionic hydrogel as the electrolyte and different electrode combinations have shown mechanically regulated potential differences. Apart from the applications mentioned above, battery-mode hydrogel-based self-powered sensors have also been reported to detect human movements, measure wrist pulse, and monitor throat vibrations [91,92,93,94]. The sensors respond to pressure and strain signals through resistance change of the ionic hydrogels under deformation. The self-power function can be achieved by either primary batteries or rechargeable batteries. Primary batteries may face the problem of chemical energy depletion, thus losing their self-power ability. Meanwhile, the coupling relationship between battery performance and sensor performance should be clarified to construct stable self-powered sensors with high sensitivity.

### 3.5. Thermoelectric Mode

Given its ubiquity and inevitability, heat can be regarded as renewable energy. Low-grade heat is released into the environment, created as a waste heat byproduct, and lost via human skin. Thermoelectric technology is the most straightforward method for converting heat directly into electrical energy. By using ionic hydrogels, it is possible to create quasi-solid-state thermoelectric cells that can convert heat to electricity. As a result, low-grade heat emitted by the human body can be harvested and utilized to power sensors [95]. Ionic thermoelectric systems are mostly based on the thermal diffusion effect of ions or the thermogalvanic effect of oxidation/reduction couples [96]. To assess the performance of thermoelectric materials, the figure of merit ZT is used, which is determined by the Seebeck coefficient (Se), electrical conductivity (σ), and thermal conductivity (κ) (ZT = *Se^2^σT*/*κ*).

**Figure 14:**
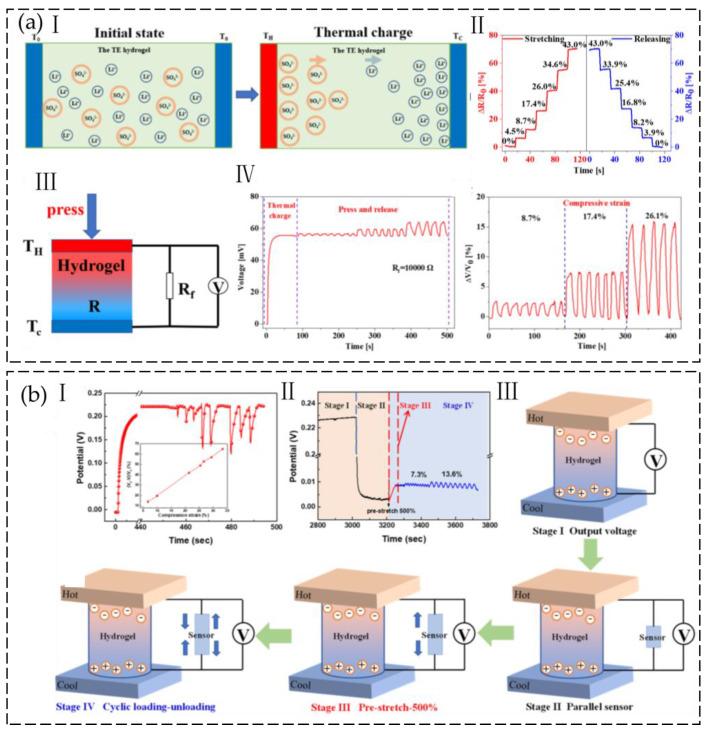
(**a**) I. Thermal voltage generation mechanism of thermodiffusion of ions. II. Relative resistance change during stretching and releasing. III. Schematic illustration of the thermo-powered ionic hydrogel strain sensor. IV. Thermal charge and voltage changes during compression and relaxation. (Reprinted with permission from [98].) (**b**) I. Voltage changes of the hydrogel at various compressive strains with a ΔT of 7.5 K. II. The voltage changes of the hydrogel sensor in parallel with thermoelectric hydrogels at various tensile strains. III. Corresponding equivalent circuit diagram. (Reprinted with permission from [99].)

The thermodiffusion of ions from the hot side to the cold side and accumulation at the cold end in the solid electrolyte will produce a thermoelectric voltage, which is called the Soret effect [97]. Ions can only build up on both sides of the electrode because they cannot pass through it. A temperature gradient causes ions to accumulate at separate electrodes, generating a thermoelectric voltage due to the net charge buildup. Chen et al. [98] prepared ionic conductive hydrogels by using polyacrylamide (PAAm) as the first crosslinked network, calcium alginate (CA) as the second crosslinked network, Li_2_SO_4_ as the ionic conductive material, and water/glycerol as the dispersion medium. A temperature difference at both ends of the ionic hydrogel causes lithium ions and sulfate ions to migrate from the hot side to the cold side, resulting in the thermodiffusion effect. Lithium ions migrate more quickly than sulfate ions in the polymer network structure because they are smaller in size. As the space between the ions grows, lithium ions will eventually gather on the cold side while sulfate ions stay on the hot side, creating a thermoelectric voltage (Figure 14(aI)). The Seebeck coefficient of the high-elasticity quasi-solid hydrogel can reach 11.5mV K^−1^. A self-powered ion-conductive hydrogel strain sensor was constructed by driving an external resistor with the thermovoltage of the ion-conductive hydrogel. When a strain is given to the ionic hydrogel at a temperature differential, the ionic hydrogel’s resistance changes, causing a proportional load–voltage shift in the fixed resistance (Figure 14(aIV)). As a result, the relative resistance change of the ionic hydrogel can be transformed into the voltage change of the fixed-load resistance, allowing the external input signal to be detected.

Zhang et al. [99] synthesized ionic hydrogels that contained NaCl through radical polymerization and metal ion complexation. The addition of CaCl_2_ created a second crosslinking network. The resulting hydrogel exhibited remarkable stretchability, withstanding up to 1500% tension when optimized. When there is a temperature differential, sodium ions and chloride ions will accumulate on the cold side and the hot side, respectively, resulting in a difference in ion concentration and a thermovoltage. Within the range of 600% to 1500%, the hydrogel sensor demonstrates a sensitivity of up to 7.01. The ionic thermovoltage and power density observed were measured to be 34.27 mV K^−1^ and 730 mW m^−2^, respectively. The hydrogel thermoelectric device, based on the ionic conductor, can be regarded as a capacitor whose open voltage decreases as the compressive strain increases (Figure 14(bI)). A self-powered strain sensor was developed using a hybrid system consisting of a hydrogel sensor and a hydrogel thermoelectric device that operates solely on thermoelectric power. The voltage changes in response to external stimuli-induced strain can be used to quantify the degree of strain, without the need for an external power source (Figure 14(bII,III)).

**Figure 15:**
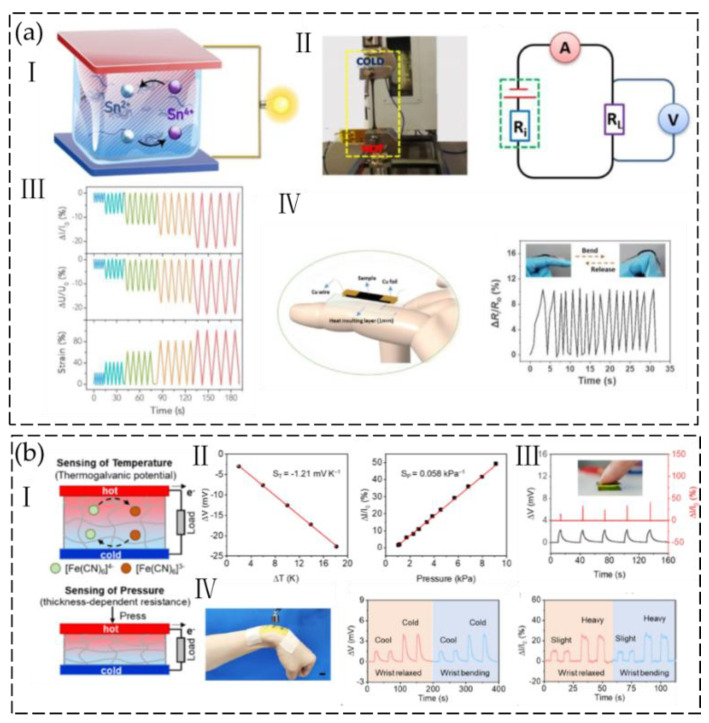
(**a**) I. Hydrogel-based TEC utilizing Sn^4+^/Sn^2+^ temperature-dependent redox reactions. II. Experimental set-up and equivalent circuit for the measurement of the self-powered strain sensor. III. Variation in current and voltage for the self-powered TEC sensor. IV. Finger movement monitoring using a self-powered strain sensor. (Reprinted with permission from [106]). (**b**) I. Schematic of temperature−pressure-sensing mechanism. II. Seebeck coefficient fitting relative current change versus pressure of the TGH sensor. III. Responses of a self-powered TGH sensor to finger touch in terms of relative current change and output thermal voltage. IV. TGH sensor array output voltage and relative current change response curves with relaxed and bending states on the human wrist. (Reprinted with permission from [100]).

For the thermogalvanic cell, redox pairs such as Fe(CN${)}_{6}^{3-}$/K_3_Fe(CN${)}_{6}^{4-}$ [100,101,102], Fe^3+^/Fe^2+^ [103], I^3−^/I^−^ [104], and SO_2−4_/SO_2−3_ [105] were introduced into ionic hydrogel electrolytes. Application of a temperature gradient across the entire cell leads to a temperature-dependent redox reaction, resulting in oxidation at the anode and reduction at the cathode of the redox couple. The reduced material then moves to the anode through convection, diffusion, and migration, where it undergoes oxidation. The oxidized material is subsequently transported back to the cathode, thereby enabling a continuous reaction. For example, Liang et al. [106] fabricated a composite hydrogel by in situ free-radical polymerization of polyacrylamide (PAAm) in the presence of acidified single-walled carbon nanotubes (a-SWCNTs). Ion exchange was then used to include the Sn^4+^/Sn^2+^ redox pair (Figure 15(aI)). The a-SWCNT can assist the transport capability of the redox ions, resulting in improved ionic conductivity. With the addition of 0.6 wt% a-SWCNTs, the interactions of redox ions with dissociated surficial groups on a-SWCNTs are optimized, resulting in improved thermoelectrochemical performance. The hydrogel-based thermoelectrochemical cells (TECs) showed remarkable flexibility and stretchability and demonstrated an outstanding Se of 1.59 ± 0.07 mV K^−1^. Their high thermoelectrochemical stability against significant mechanical stretching and deformation was demonstrated when they maintained relatively constant ionic conductivity and Seebeck coefficient under 100% strain. A self-powered sensor was constructed by connecting a TEC in series with a load resistor. The current in the circuit and the voltage generated on the load were monitored in real time using an ammeter and a voltmeter, respectively (Figure 15(aII)). When the applied strain increases, the internal resistance of the TEC increases as well. Consequently, both the loop current and the voltage generated on the load resistor decrease (Figure 15(aIII)). The gauge factor can reach 0.4 when the strain is below 20% and 0.61 when the strain is between 20% and 100%. The TEC sensor can be self-powered by using the skin surface as a heat source and can be used for in situ monitoring of human motions of finger bending (Figure 15(aIV)). 

Fu et al. [100] developed a self-powered temperature–pressure dual-sensing electronic skin based on thermogalvanic hydrogels (TGHs) by introducing the redox couple K_4_Fe(CN)_6_/K_3_Fe(CN)_6_ into a polyacrylamide hydrogel (Figure 15(bI)). Due to the combination of the thermogalvanic effect and piezoresistive effect of TGH, temperature and pressure stimuli can be converted into voltage and current signals, allowing both parameters to be simultaneously detected. The thermogalvanic hydrogel demonstrates an equivalent Seebeck coefficient of −1.21 mV K^−1^ and a pressure sensitivity of 0.056 kPa^−1^ (Figure 15(bII)). Simultaneously applying temperature and pressure stimuli to the TGH sensor results in an output voltage that not only detects temperature differences but also acts as an internal power source to drive pressure sensing (Figure 15(bIII)). Additionally, the sensing of pressure and sensing of temperature do not conflict with one another. The multifunctional sensor can be utilized to precisely record tactile information on human skin and spatial perception when combined with unit array integration (Figure 15(bIV)). Similarly, Li et al. [101] developed a thermogalvanic device made of a poly(vinyl alcohol)/gelatin (PVA/GEL) dual-network hydrogel with Fe(CN${)}_{6}^{3-}$/K_3_Fe(CN${)}_{6}^{4-}$ as a redox pair. The limited hydrogen bonding between water molecules in the H_2_O/GL (glycerin) solvent endows the hydrogel with outstanding low-temperature durability and antidrying capacity. The prepared thermogalvanic hydrogel delivers a Seebeck coefficient of −1.21 mV K^−1^. The gel was attached to a mask to create a self-powered sensor for monitoring respiration.

These findings show that ionic hydrogels with excellent ionic thermal diffusion or thermogalvanic effects are possible options for self-powered performance of a hydrogel wearable tactile sensor. However, challenges remain in the low energy conversion efficiency and thermoelectric figures of merit for thermoelectric hydrogels. Further systematic and in-depth investigations are necessary to explore potential technologies that can enhance the Seebeck coefficient for the purpose of utilizing them in self-powered electronics.

## 4. Conclusions and Perspective

Hydrogels are a significant family of materials with highly adjustable physical and chemical characteristics. Ionic hydrogels have gained significant interest in the fields of health monitoring and human–computer interaction due to their high structural similarity to natural soft tissues and design flexibility. The research progress of ionic hydrogel self-powered sensors in recent years has been introduced. The utilization of ionic hydrogels in tactile sensors has provided them with excellent stretchability and a wide working range. Furthermore, it is possible to attain a high-performance tactile sensor through performance optimization and structural design based on ionic hydrogel technology. In order to endow a sensor with self-powered characteristics, it is necessary to obtain energy from the surrounding environment according to different application scenarios. Some outstanding developments of sensors working in different modes have been summarized. The progress presents novel opportunities and advancements in the domains of motion monitoring, electronic skin, and soft robotics.

Although there have been many advancements made in the field of self-powered sensors, there are still some problems that are hoped to be resolved with continued work to make them more practical. Most importantly, it is still crucial to improve the sensitivity and energy-harvest efficiency of ionic self-powered sensors. For their practical use as electrodes in triboelectric sensors, improvement is still required in terms of ionic conductivity and long-term stability. The self-power supply ability of the ionic hydrogel sensors working in piezoionic, ionic diode, and thermoelectric modes mainly come from the directional movement or concentration gradient of ions in the hydrogel. So, efforts should be focused on the molecular and structure design of the ionic hydrogel to enlarge the differential of ion transmission and concentration gradient. In addition, it is necessary to clarify the coupling relationship between external stimulation and ion transmission, that is, sensing performance and self-powered performance. For sensors working in battery mode, the Faraday reaction exists at the interface between the electrode and ionic gel, and the stability of the ionic hydrogel should be considered. 

Currently, ionic hydrogel devices are often made directly from bulk hydrogel, with hand assembly serving primarily as the patterning and integration step. The functionality of a single device will be improved by the miniaturization, microfabrication, and scalable manufacture of hydrogel devices.

Ionic hydrogel sensors are mostly created with a single- or dual-operation modality. The human skin, however, is capable of multimodal sensation. In order to create new kinds of multimodal sensors, it is worthwhile to investigate innovative designs for hydrogel materials and structures.

Despite the fact that the stability limitations resulting from the drying and freezing of hydrogels have been resolved, the trade-off with decreased ionic conductivity should be optimized. Moreover, the challenges for stability also include interferences in monitoring human activity from the external environment, such as varying pH conditions, humidity, temperature, and gas environments.

In conclusion, ionic hydrogels have been shown to be exceptional materials for fabricating wearable sensing devices. It is anticipated that self-powered ionic hydrogel sensors will advance quickly and play a crucial part in the development of the next generation of electronic systems, making e-skin technology applicable to both business and everyday life.

## Data Availability

Data sharing not applicable.
